# Functions of *thga1* Gene in *Trichoderma harzianum* Based on Transcriptome Analysis

**DOI:** 10.1155/2016/8329513

**Published:** 2016-09-08

**Authors:** Qing Sun, Xiliang Jiang, Li Pang, Lirong Wang, Mei Li

**Affiliations:** State Key Laboratory for Biology of Plant Diseases and Insect Pests, Institute of Plant Protection, Chinese Academy of Agricultural Sciences, Beijing 100081, China

## Abstract

*Trichoderma *spp. are important biocontrol filamentous fungi, which are widely used for their adaptability, broad antimicrobial spectrum, and various antagonistic mechanisms. In our previous studies, we cloned* thga1* gene encoding G*α*I protein from* Trichoderma harzianum* Th-33. Its knockout mutant showed that the growth rate, conidial yield, cAMP level, antagonistic action, and hydrophobicity decreased. Therefore, Illumina RNA-seq technology (RNA-seq) was used to determine transcriptomic differences between the wild-type strain and* thga1 *mutant. A total of 888 genes were identified as differentially expressed genes (DEGs), including 427 upregulated and 461 downregulated genes. All DEGs were assigned to KEGG pathway databases, and 318 genes were annotated in 184 individual pathways. KEGG analysis revealed that these unigenes were significantly enriched in metabolism and degradation pathways. GO analysis suggested that the majority of DEGs were associated with catalytic activities and metabolism processes that encode carbohydrate-active enzymes, secondary metabolites, secreted proteins, or transcription factors. According to the functional annotation of these DEGs by KOG, the most abundant group was “secondary metabolite biosynthesis, transport, and catabolism.” Further studies for functional characterization of candidate genes and pathways reported in this paper are necessary to further define the G protein signaling system in* T. harzianum*.

## 1. Introduction

*Trichoderma *is an important fungal genus, whose species exhibit favorable properties, such as diverse mechanisms of antagonistic action, a broad spectrum of activity in plant disease prevention and control, survival under unfavorable conditions, and environmental friendliness.* Trichoderma harzianum* is one of the most commercially used biofungicides, particularly* T. harzianum* T22 and T39 [1]. Growth, conidiation, secondary metabolism, and mycoparasitism are all important processes that contribute to biofungicidal property [2]. An enhanced understanding of the signal regulatory mechanism in* T. harzianum* is necessary to further explore the fungi's extraordinary biocontrol potential.

G protein-mediated signal transduction system is an important transmembrane signaling system in eukaryotic cells. It plays a key role in the regulation of cellular reactions and the transmission of extracellular signals to cells. The role of G protein in the transmission of external stimuli has been studied in detail in genetic fungi models, such as* Neurospora, Penicillium* [3], and* Aspergillus* [4]. Heterotrimeric G protein is composed of *α*,  *β*, and *γ* subunits; each subunit is encoded by independent genes. Among the G protein subunits, the G*α* subunits were found more frequently and reported to regulate vegetative growth, conidiation, and the mycoparasitic responses in fungi [5]. However, current observations showed that a particular G*α* subunit may have different functions in different fungal species [6]. G*α* subunits are classified into three groups according to conserved motif sequences [7], and several subgroup G*α*I subunits from* T. atroviride* and* T. virens* have been studied [4, 7]. G*α* from* T. atroviride*,* tga1*, was reported to negatively regulate conidiation but had no effect on hyphal growth [8].* TgaA* is a homology gene of* tga1* from* T. atroviride*, which does not influence growth and conidiation in* T. virens*. The* ΔtgaA* mutants grow normally and sporulate like the wild type but possess a reduced ability to colonize* Sclerotinia sclerotiorum*, whereas they are fully pathogenic against* Rhizoctonia solani* [4].

In our previous study, a subgroup I G*α* gene (*thga1*) was cloned from the* T. harzianum* Th-33 genome. Results showed that THGA1 has the same amino acid sequence as that of TGA from* T. atroviride* but with different functions. Compared with the wild-type Th-33, the* Δthga1 *mutants changed significantly in biological characteristics and physicochemical properties. In particular, the hyphal growth rate dropped by about 40%, conidiophore branches became sparse, secondary branch and phialide numbers reduced, conidiation was delayed for about 20 h, and conidia yield declined by about 300-fold. In addition, the hydrophobicity of the mutants weakened, intracellular cAMP levels decreased by about 50%, and the inhibition of the mutant against plant pathogen of* R. solani* reduced significantly. The results showed that the* thga1* gene positively affected the growth, conidial production, hydrophobicity, and antagonism of* T. harzianum* to* R. solani*.

The present study characterized the genes under- or overexpression in the mutant compared to WT associated with the G*α* subunit,* thga1*, using next-generation RNA sequencing (RNA-seq) technology, to gain insight into the regulatory mechanism of G*α* subunits* thga1* in Th-33. This study is the first initiative to use RNA-seq for identifying differentially expressed genes (DEGs) to clarify the function of G*α* in* Trichoderma* by genome-wide transcriptional analysis of hyphal cells of the wild-type Th-33 and its* Δthga1 *mutants. The results may reveal that G*α* regulated a series of biological processes as well as new targets of* thga1* function.

## 2. Materials and Methods

### 2.1. Strains and Culture Media

The wild-type* T. harzianum* Th-33 was isolated from soil samples in the Beijing region as described previously. The* Δthga1* knockout mutant was created with hygromycin B resistance by homologous recombination and then purified by isolation of single conidia [9]. Mutant colony was identified by southern hybridization. The fungi were grown on potato dextrose agar (PDA) at 28°C for conidia production. The PDA medium was covered with cellophane for mycelia collection. Conidial suspensions were prepared by adding sterilized distilled water to the PDA plates. The conidial suspensions were inoculated on the PDA medium covered with cellophane and cultured for 32 h prior to conidia formation at the tip of the mycelia. The mycelia were then scraped off the cellophane, washed with cold distilled water, frozen in liquid nitrogen, and ground to a fine powder. Equal mixture of three biological samples was sequenced.

### 2.2. Preparation of the cDNA Library and Illumina Sequencing for Transcriptome Analysis

Total RNA was extracted using Trizol reagent (Invitrogen, CA, USA) according to the manufacturer's instruction. The RNA quality and quantity were determined using an Agilent 2100 Bioanalyzer. The Qubit RNA Assay Kit was used for accurate quantification of the initial total RNA. After total RNA extraction and DNase I treatment, magnetic beads with oligo (dT) were used to isolate mRNA. The mRNA was mixed with the fragmentation buffer to promote fragmentation into short segments. Subsequently, cDNA was synthesized using SuperScript II reverse transcriptase following the manufacturer's protocol. The short fragments were connected with adapters. The suitable fragments were selected as templates for PCR amplification. During the QC steps, a 2100 Bioanalyzer High Sensitivity DNA chip was used for quantification and qualification of the sample library. RNA libraries were sequenced by paired-end mode using an Illumina HiSeq2000 system.

### 2.3. Sequence Alignment

Clean reads were obtained after removal of low quality reads, reads with adaptors, and reads with unknown nucleotides larger than 5%. All reads were deposited in the National Center for Biotechnology Information (NCBI) database and can be found under accession number SRS823675. The reads were mapped to the reference genome [10] of* T. harzianum *Th-33 using TopHat (Version: 2.0.11) program [11].

### 2.4. Expression Analysis

Expression values were obtained by calculating the fragments per kilobase of transcript per million mapped reads, and differential gene expression was analyzed using the Cufflinks program [12]. Genes differentially expressed with more than twofold changes and at *p* values less than 0.05 were identified as DEGs. The threshold of the *p* value in multiple tests was determined by the value for the false discovery rate (FDR) [13]. We used “FDR ≤ 0.001 and the absolute value of log_2_ fold change (log_2_ FC) ≥ 2” as the threshold to assess the significance of gene expression differences.

### 2.5. Functional Annotation of* T. harzianum* Transcriptome and Classification of DEGs

The* T. harzianum* transcriptomes were compared against amino acid sequences available at the UniProt database using BLASTx algorithm. For each query sequence, the associated hits were searched for their respective gene ontology (GO) and Kyoto Encyclopedia of Genes and Genomes (KEGG) results. The highest bit score was selected, with *E*-value threshold of 1*e*201. Protein families were classified by searching the assembled transcripts against Pfam and InterProScan. Potential transmembrane domains of the G protein-coupled receptors (GPCRs) selected from InterProScan and Pfam analysis were predicted using transmembrane domain prediction tool TMHMM v2.0. GPCRs predicted to contain the seven-transmembrane domain were used for further analysis. We used the CAZy database to annotate the carbohydrate-active enzymes (http://www.cazy.org/). We annotated transcription factors (TFs) of DEGs based on protein sequence homology using the Fungal Transcription Factor Database (http://ftfd.snu.ac.kr/). The amino acid sequences of DEGs were further analyzed to predict secreted proteins. Sequences smaller than 70 amino acids were not considered for further analysis. The remaining sequences with positive SignalP prediction for signal peptide cleavage site at the N-terminal region between 10 and 40 amino acids, without any transmembrane region, were selected as the candidate secreted proteins. Secreted proteins with lengths less than 200 amino acids and cysteine content more than 4% of the protein were identified as small molecule cysteine-rich secreted proteins (SSCPs).

### 2.6. Validation of RNA-Seq Results by Quantitative Real-Time RT-PCR (qRT-PCR)

To validate the expression profiles of the assembled genes obtained through sequencing data analysis, qRT-PCR was performed for selected genes. Genes were randomly selected, and four reference genes were used, namely, beta-tubulin 1, actin, transcription elongation factor, and ubiquitin-conjugating enzyme (UCE). Primers used in qRT-PCR were designed using Primer Express Software v2.0 (see S2 Table in Supplementary Material available online at http://dx.doi.org/10.1155/2016/8329513). Total RNA (2 *μ*g) from each sample was reverse transcribed into cDNA in the presence of oligo (dT) primer in a volume of 12.5 *μ*L. The synthesized cDNA was used as a template for qRT-PCR. Reactions were performed in the ABI StepOne Plus Real-Time PCR System (Applied Biosystems). Each reaction (20 *μ*L) contained 10 *μ*L of 2x SYBR Green PCR mix (QIAGEN), 1 *μ*L of forward and reverse primers (10 pM/*μ*L each), 1 *μ*L of cDNA template, and 7 *μ*L of nuclease-free water. PCR cycling conditions were 2 min at 95°C (1 cycle) and 10 s at 94°C, followed by 10 s at 60°C and a melting curve of 40 s at 72°C (40 cycles). PCR cycles for negative controls without templates were carried out concurrently. All qPCRs for each gene used three biological replicates, with three technical replicates per experiment. The average threshold cycle (Ct) was calculated using the 2^−ΔΔCt^ method [14].

### 2.7. Availability of Supporting Data

Sequences have been deposited at the Sequence Read Archive (SRA) of the NCBI under BioProject number PRJNA272748. Raw sequence reads can be found in http://trace.ncbi.nlm.nih.gov/.

## 3. Results and Discussion

### 3.1. Illumina Sequencing

We got 17 transformants with stable genetic characteristics. The homologous recombination events have been confirmed by southern hybridization. A probe corresponding to part of the* thga1* gene coding region gave no signal on a southern blot of the deletion mutants, while the hygromycin resistance cassette was detected for the mutants but not for the wild type (data not shown). We selected one mutant with obvious phenotypic differences to be studied. Two cDNA libraries were constructed and subjected to Illumina deep sequencing. Approximately 2,838,821,746 and 3,242,007,080 bp clean reads were generated for the wild type and the mutant 1-1, respectively (Table 1). The reads were then compared against the genome of Th-33, which was sequenced and submitted to SRA (http://trace.ncbi.nlm.nih.gov/Traces/sra/). The whole genome of* Trichoderma harzianum* Th-33 was sequenced on a Hiseq2500 instrument. A total of 196 scaffolds were assembled and 10849 genes were predicted with an average length of 1776 bp (GenBank number: PRJNA272949). The unigenes were used for transcriptome analysis in this study. The overall mapping rate was about 95%, indicating that subsequent analysis can be performed.

### 3.2. DEGs Regulated by* thga1*

RNA-seq technology was used to investigate the transcriptional changes between the wild-type Th-33 and the mutant regulated by* thga1*. Comparisons between the gene expression of* thga1* mutant and wild-type Th-33 showed that 888 genes were differentially expressed, including 427 upregulated and 461 downregulated genes. Among the 20 significantly upregulated genes (S1 Table), 13 genes exhibited defined functions, including six metabolism-related genes and two genes predicted to encode enzymes. Among the significantly downregulated genes (S1 Table), seven were metabolism-related genes and four were catalytic activity genes. The changes in expression were evidently connected to metabolism. Hence,* thga1* might regulate a series of biological processes through metabolism.

### 3.3. Real-Time qRT-PCR Analyses

**Figure 1:**
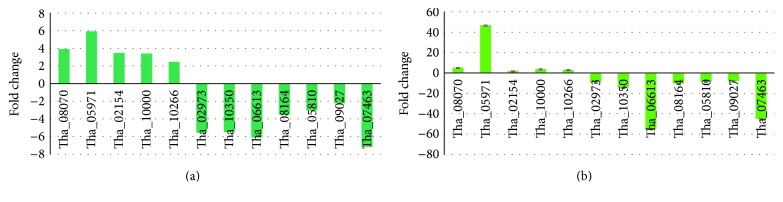
Transcriptome validation. (a) DEG data in transcriptome analysis. The fold changes of the genes were calculated as the log_2_ value of each 1-1/Th-33 comparison and are shown on the *y*-axis. (b) The qRT-PCR analysis of gene expression data. Expression ratios of selected genes in 1-1 compared to Th-33.

To verify the quality of the assembly, cDNA fragments of 15 randomly selected unigenes were amplified using unigene-specific primers (S2 Table) and then sequenced. Four reference genes (beta-tubulin 1, actin, transcription elongation factor, and UCE) were used as endogenous control. Among the reference genes, UCE remained constant in all treatments and showed the best performance in qRT-PCR analysis using Best Keeper program. Expression patterns of the tested genes are shown in Figure 1. Three genes with infinite fold changes in the RNA-seq results were not shown in the figure, as they cannot be shown in the bar chart. Expression patterns determined by real-time qRT-PCR were consistent with those obtained by RNA-seq, thereby confirming the accuracy of the RNA-seq results reported in this study.

### 3.4. Pathway Functional Enrichment Analysis of DEGs

**Figure 2:**
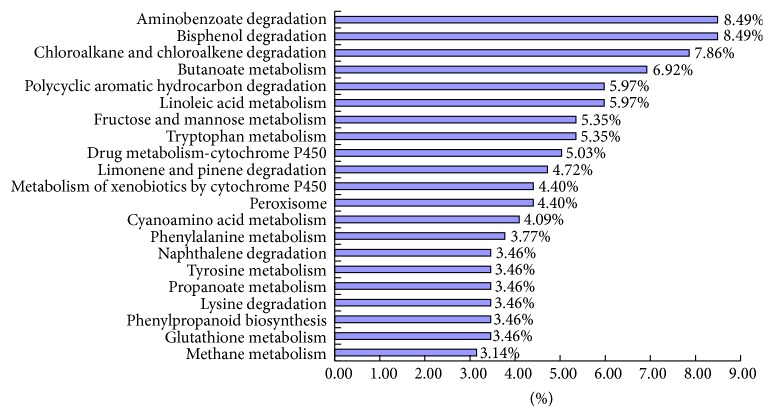
Pathway enrichment analysis of DEGs. The percentage of differentially expressed genes involved in KEGG pathways. Only the top 21 most abundant EC and KEGG pathways are represented.

In our research, 318 differentially expressed unigenes were assigned to 184 KEGG pathways. A summary of the findings is presented in S3 Table. Among the pathways identified, the metabolic pathways, especially the secondary metabolic pathways, were found to be the most active. “Bisphenol degradation” (27 unigenes, 8.49% of sequences), “aminobenzoate degradation” (27 unigenes, 8.49% of sequences), “chloroalkane and chloroalkene degradation” (25 unigenes, 7.86% of sequences), and “butanoate metabolism” (22 unigenes, 6.92% of sequences) were the dominant pathways (Figure 2). Thus,* thga1* may affect growth, conidiation, and antagonism through metabolism. The DEG catalogue provided a comprehensive understanding of the gene transcription profiles of* T. harzianum* missing the* thga1* gene and offered a valuable foundation for the screening of G protein-mediated pathways.

### 3.5. GO Enrichment Analysis

**Figure 3:**
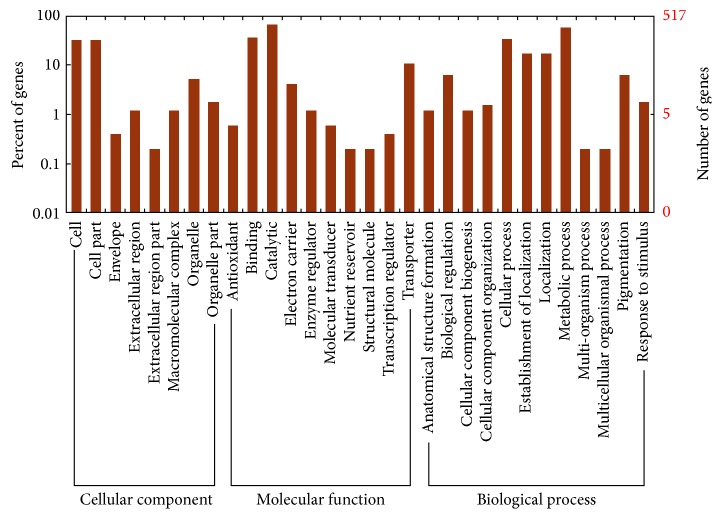
Gene ontology terms for differentially expressed genes. Most differentially expressed genes were grouped into three major functional categories: cellular component, molecular function, and biological process.

Blast2GO [15] program was used for GO analysis. The program extracted the GO terms associated with the homologies identified by BLAST and returned a list of GO annotations, which were presented as hierarchical categories of increasing specificity. GO enrichment analyses were performed using Fisher's exact test with multiple testing corrections and an FDR of 0.05. A total of 517 DEGs were categorized into 707 functional groups in three main categories, namely, “cellular component,” “molecular function,” and “biological process” (S4 Table and Figure 3). Some unigenes were assigned to multiple categories of GO terms, whereas others could not be assigned to a given GO term. In the biological process category, “metabolic process” (381, 73.69%), “cellular process” (201, 38.87%), “single-organism process” (115, 22.24%), “localization” (100, 19.34%), and “establishment of localization” (99, 19.15%) were the most abundant terms. In the molecular function category, genes associated with “catalytic activity” (351, 67.89%), “binding” (191, 36.94%), and “transporter activity” (54, 10.44%) were the most abundant. In the cell component category, “membrane” (124, 23.98%) and “membrane part” (100, 19.34%) were the most abundant terms. These findings indicated that the main changes in expression between the wild-type Th-33 and the* thga1* mutant were those related to membrane parts, metabolic processes, and catalytic activities.

### 3.6. KOG Function Classification

**Figure 4:**
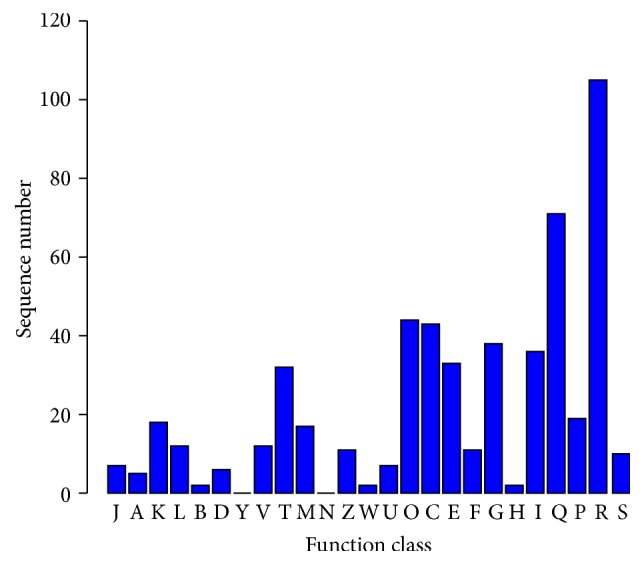
KOG function classification of the differentially expressed genes. J: translation; ribosomal structure; biogenesis. A: RNA processing and modification. K: transcription. L: DNA replication; recombination; repair. B: chromatin structure and dynamics. D: cell cycle control; cell division; chromosome partitioning. Y: nuclear structure. V: defense mechanisms. T: signal transduction mechanisms. M: cell wall/membrane/envelope biogenesis. N: cell motility. Z: cytoskeleton. W: extracellular structures. U: intracellular trafficking; secretion; vesicular transport. O: posttranslational modification; protein turnover; chaperones. C: energy production and conversion. E: amino acid transport and metabolism. F: nucleotide transport and metabolism. G: carbohydrate transport and metabolism. H: coenzyme transport and metabolism. I: lipid metabolism. Q: secondary metabolites biosynthesis; transport; catabolism. P: inorganic ion transport and metabolism. R: general function prediction only. S: function unknown.

DEGs were also searched against the KOG database for functional prediction and classification. Notably, out of 888 distinct DEGs, 463 could be functionally classified into 23 molecular families, which was consistent with the approximately 50% KOG annotation rate of the Frozen Gene Catalogue (S5 Table and Figure 4). The largest number of unigenes focused on “the general function of prediction (19.3%),” whereas the next largest groups were noted in “secondary metabolite biosynthesis (13.1%), transport, and catabolism (13.1%),” followed by “posttranslational modification; protein turnover; chaperones (8.1%),” “energy production and conversion (7.9%),” “carbohydrate transport and metabolism (7.0%),” “lipid metabolism (6.6%),” “amino acid transport and metabolism (6.1%),” and “signal transduction mechanisms (5.9%).” The three smallest groups were included in “chromatin structure and dynamics (0.37%),” “extracellular structures (0.37%),” and “coenzyme transport and metabolism (0.37%).” Genes involved in intermediary metabolism (i.e., of carbohydrates, amino acids, and lipids) comprised a significant portion within both up- and downregulated genes.

### 3.7. Genes Related to G Protein Signal Pathway

G protein transduced signals received by the heptahelical GPCRs from outside the cell influence numerous regulatory pathways via their respective effectors, which in turn affect the activities of secondary messengers [16, 17]. Three G*α*-coding genes, one beta, and one gamma subunit genes were found in the transcriptomes and the genome of Th-33, which corresponded well with the data of* T. virens* [18],* Neurospora crassa* [19], and many other filamentous fungi [6].* Thga1*, the subgroup I G*α* gene, was not expressed in the* Δthga1* mutant. Another G*α* gene, a subgroup II* tgaB* homologous gene in* T. virens* [4], exhibited downregulated expression (twofold), whereas the third G*α* gene *β* and G*γ* gene were not differentially expressed. These results showed that knockout of* thga1* caused the downregulated expression of another G*α* gene.

The signals transmitted through a heterotrimeric G protein signaling cascade originate from the activation of plasma membrane-localized GPCRs. The identification and characterization of GPCRs will provide insights into how* Trichoderma* communicates with G protein and downstream genes. Thirty-nine GPCRs or putative GPCR genes were found in the transcriptome of Th-33. Six of them were differentially expressed in the mutant (five of them belong to the PTH11-type; Table 2). PTH11-type GPCRs were reported to influence light responsiveness, glycoside hydrolase gene transcription, sexual development [20, 21], surface recognition, and pathogenicity [22]. PTH11 GPCR genes were reported to be upregulated in the mycoparasite* Coniothyrium minitans* during colonization of* S. sclerotiorum* [23]. In the current study, five PTH11-type GPCR-encoding genes were downregulated in the mutant. This observation was consistent with the phenomenon that the inhibitory effect of the mutant against* R. solani* decreased significantly compared with that of the wild-type Th-33.

cAMP acts as a secondary messenger for morphogenic signals in both prokaryotes and eukaryotes, and it is generated by adenylate cyclase and degraded by phosphodiesterase [24]. In* T. virens*, the intracellular cAMP levels can be regulated by an adenylate cyclase gene,* tac1* [25], and it has been reported to influence conidiation [26]. In* Aspergillus nidulans*, a G*α*-subunit* GanB* mediates a rapid and transient activation of cAMP synthesis in response to glucose during the early period of germination [27]. In our previous study, intracellular cAMP levels in the* Δ*t*hga1* mutant decreased by about 50% of that in the wild-type Th-33. According to the transcriptomes of the* Δthga1* mutant and the wild-type Th-33, the adenylate cyclase-encoding gene was not expressed differentially, whereas the cAMP phosphodiesterase gene was found to be upregulated in the mutant. These findings indicated that the decrease in intracellular cAMP levels was caused by the increase in cAMP phosphodiesterase activity and not by the decrease in adenylate cyclase activity. This study showed that the G protein *α* subunit in* Trichoderma *may be involved in the mechanisms regulating intracellular cAMP levels.

### 3.8. Carbohydrate Activity Enzymes (CAZymes)

**Figure 5:**
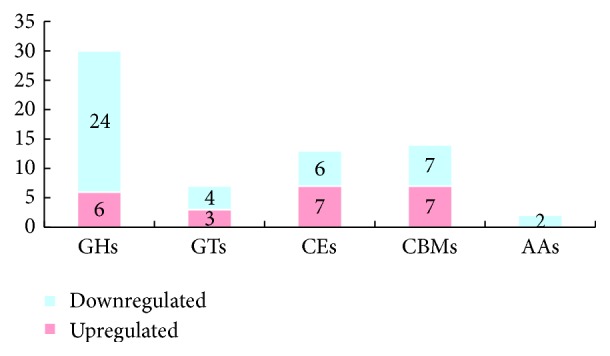
Numbers of carbohydrate-active enzyme modules in the differentially expressed genes.

CAZymes in fungi are correlated with their nutritional modes and infection mechanisms [28]. Various CAZyme genes exist in the Th-33 genome, of which 66 differentially expressed CAZyme genes were identified in the Th-33 transcriptome, including 30 genes from the glycoside hydrolase family (GH), 14 from the auxiliary activity family, 13 from the carbohydrate esterase family, 7 from the glycosyltransferase family, and 2 from the carbohydrate-binding module family (S6 Table). In addition, the number of downregulated CAZyme genes (43 genes) in the mutant was greater than that of upregulated genes (23 genes) (Figure 5). The GH family has been described to play a central role in mycoparasitism in* Trichoderma*. For example, GH18 family is highly expanded in* T. atroviride* and* T. virens* during mycoparasitism [29]. GH16 was reported to be upregulated during the mycoparasitic reaction of* T. atroviride* against* R. solani* [30]. In the present study, the inhibition of the* Δthga1* mutant* T. harzianum* against* R. solani *and* Phytophthora capsici* was reduced significantly, and the mycelia of* Δthga1* could not cover and parasitize the pathogenic fungi's mycelia in confrontation tests. The heterotrimeric G protein pathway is crucial in the interconnections of nutrient signaling in* Trichoderma* [21]. Results implied that the knockout of* thga1* caused the decrease in the activities of the CAZymes, which consequently influenced nutrient condition, further retarded the growth rate, and decreased the mycoparasitic ability of* Trichoderma*.

### 3.9. TFs

**Figure 6:**
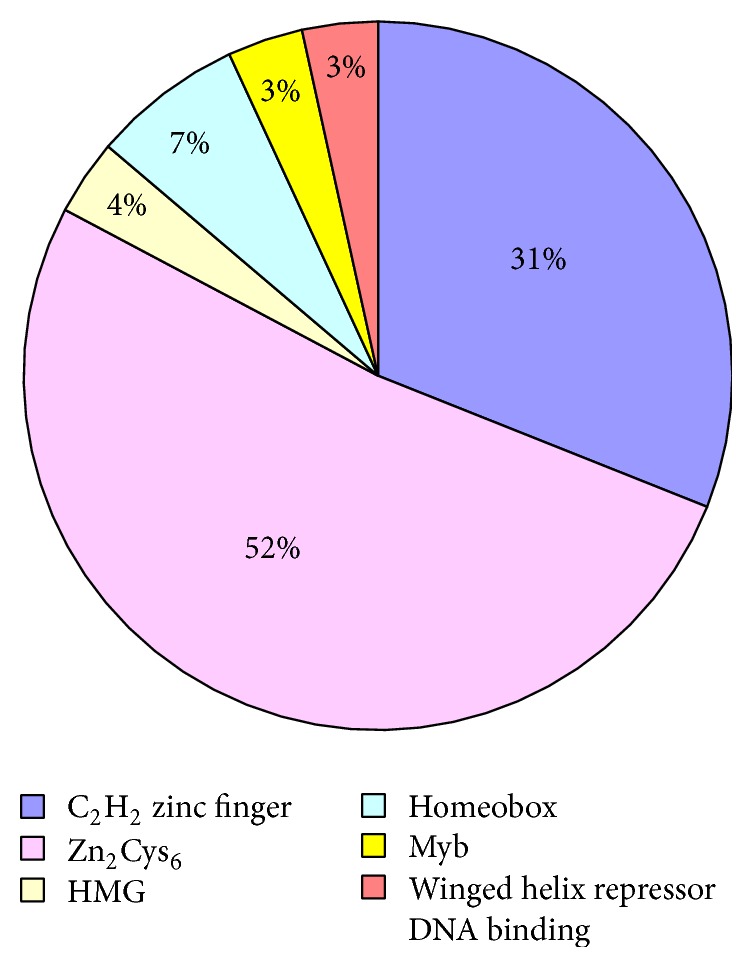
Percentage of different TFs in the DEGs.

Twenty-nine TFs were identified in the* Δthga1* mutant and compared with that of the wild-type Th-33. Of these TFs, 15 were upregulated and 14 were downregulated TFs (S7 Table). Among those differentially expressed TFs, the Zn_2_Cys_6_ family (53%) was the most dominant TF family, followed by the C_2_H_2_ zinc finger (31%) TF family (Figure 6). The functions of most TFs in fungi appear to be highly diverse and remain rather unclear. Of the known TF families, the Zn_2_Cys_6_ TF family comprises TFs that are fungal-specific and dominant [31]. These TFs are involved in primary and secondary metabolism, drug resistance, and meiotic development (http://ftfd.snu.ac.kr/). BglR, a Zn_2_Cys_6_-type TF in* T. reesei*, can increase the expression of the *β*-glucosidase gene [32]. C_2_H_2_ zinc finger in fungi is involved in pathogenicity, carbon catabolite repression, acetamide regulation, and differentiation of ascogenous or fruiting body. GipA in* A. fumigatus* was reported to be involved in secondary metabolic processes [33]. In* A. nidulans*, brlA defined the central regulatory pathway that controls conidiation-specific gene expression, but no orthologue of brlA in* Trichoderma* conidiophores currently exists [34]. TFs can control gene transcription rates and are key mediators of cellular function [35]. In the present report, differentially expressed TFs might play key roles in cell processes in* Trichoderma* under the control of the G protein signal pathway. Understanding the regulatory mechanisms of these TFs and their functions could provide valuable insight into the signal control pathways of cell processes in* Trichoderma*.

### 3.10. Secretome of* T. harzianum*

A total of 137 genes were annotated as secreted proteins, including 51 upregulated and 86 downregulated genes among the DEGs. Secreted proteins play an important role in the process of cell signaling, cell proliferation, differentiation, apoptosis regulation, development, and other important biological processes. In* Botrytis cinerea*,* bcg1* encodes *α* subunits of heterotrimeric GTP-binding proteins; the* bcg1* null mutants differ in colony morphology from the wild-type strain, which do not secrete extracellular proteases [36]. SSCPs comprise one of the largest groups of proteins secreted by* Trichoderma* [29]. Hydrophobins, which are probably the most widely known SSCPs, are found on the outer surfaces of cell walls of hyphae and conidia.* Trichoderma* genomes encode an unusually large number of hydrophobins, possibly for the purpose of providing flexibility in surface properties needed for conidiation, mycoparasitism, and interaction with plant roots [2, 37]. In the present report, two SSCPs were found among the DEGs, and these genes were downregulated in the mutant strain, which might account for the phenomenon of reduced hydrophobicity in the mutant. This was in accordance with the report that G alpha subunit can mediate fungal conidiation and hydrophobin synthesis in* Cryphonectria parasitica* [38]. Furthermore, SSCPs were shown to be highly expressed in* T. atroviride* during colonization of* R. solani,* which suggested a potential role in mycoparasitism [30]. These genes may also explain the significant reduction in the inhibition of the mutant against the plant pathogen* R. solani.*

### 3.11. Secondary Metabolism

KOG functional classification of DEGs revealed that the number of genes involved in secondary metabolite biosynthesis, transport, and catabolism was 71, in which cytochrome P450s (CYPs) accounted for nearly 24% (S8 Table). CYPs play an important role in the physiology of fungi and are involved in the biosynthesis of secondary metabolites (SMs) and in detoxification [39]. Fungi produce a wide range of SMs that are not directly essential for growth and yet have important roles in signaling, asexual conidiation [40], and interactions with other organisms [41–43].* Trichoderma *spp. are a rich source of SMs, such as nonribosomal peptides, polyketides, terpenoids, and pyrones. Although a clear correlation exists between conidiation of the fungus and the secretion of antifungal metabolites in the* T. atroviride* parental strain, its type G*α*III gene* Δtga3* mutants were fully impaired in the production of peptaibols despite exhibiting a hypersporulating phenotype [5]. The results in the current report implied that* thga1* regulated certain secondary metabolism processes, and analysis of SMs should be carried out for further identification.

## 4. Conclusion

Overall, this study identified transcripts that were possibly involved in several important molecular and cellular functions of* T. harzianum*. However, additional studies aimed at the functional characterization of the genes reported herein will aid in further defining the pathways mediated by G protein in* T. harzianum*. An enhanced understanding of the expression profiles of these genes could improve* T. harzianum* performance, either by predicting the regulation of the genes involved in sporulation or by improving their use in biotechnology processes.

## Supplementary Material

The supplementary material contains: list of Trichoderma harzianum genes with significantly altered expression (S1 Table), primers used in validation experiments (S2 Table), summary of differentially expressed genes (DEGs) assigned to the KEGG database (S3 Table), number of DEGs assigned to GO classification (S4 Table), number of DEGs assigned to KOG function classification (S5 Table), DEGs coding for carbohydrate activity enzymes, arranged by family (S6 Table), genes coding for transcription factors, arranged by family (S7 Table), DEGs involved in secondary metabolite biosynthesis, transport, and catabolism (S8 Table). All these documents are in .xls format.

## Figures and Tables

**Table 1:** Summary of Illumina transcriptome sequencing data for the strains included in this study.

Sample	Read	Length	QV	Reads number	q20 (%)	q30 (%)	Yield
Th-33	Reads 1	101	36	14053573	98.24	94.96	1419410873
	Reads 2	101	35	14053573	96.89	93.09	1419410873

1-1	Reads 1	101	36	16049540	98.35	95.24	1621003540
	Reads 2	101	35	16049540	96.53	92.46	1621003540

**Table 2:** Differentially expressed genes related to the G protein signal pathway.

Gene_id	Th-33	1-1	log_2_ (fold change)	*p* value	Annotation
Tha_09027	215.43	46.28	−2.22	0.0475	G protein alpha subunit TgaB
Tha_10266	11.00	60.75	2.47	0.0225	Cyclic AMP phosphodiesterase
Tha_08164	216.24	19.09	−3.50	0.0019	GPCR, PTH11-type
Tha_04234	107.24	11.21	−3.26	0.0053	GPCR, PTH11-type
Tha_05810	217.59	26.05	−3.06	0.0051	GPCR, PTH11-type
Tha_07855	75.77	14.01	−2.43	0.0121	G protein-coupled receptor
Tha_07047	35.10	6.85	−2.36	0.0308	GPCR, PTH11-type
Tha_06854	2.35	0	Inf	0.0078	GPCR, PTH11-type
